# 

**Published:** 1869-03

**Authors:** 


*The Baltimore College of Dental Surgery*—The TWENTY NINTH Annual Commencement of this College will be held on Wednesday evening, March 3d, at the Concordia Opera House.

The Valedictory address will be delivered by Professor E. Lloyd Howard, M. D. The Alumni and friends of the institution are cordially invited to attend.

